# Indication and diagnostic method selection for invasive prenatal genetic testing – review of the literature and conclusions of the Austrian consensus conference on prenatal genetic testing

**DOI:** 10.1515/medgen-2026-2012

**Published:** 2026-04-16

**Authors:** Sarah Verheyen, Barbara Pertl, Sophie Bierbaumer, Claudius Fazelnia, Sarah Francesca Jauch, Philipp Klaritsch, Elisabeth Krampl-Bettelheim, Stefan Kühberger, Theresa Reischer, Ricarda Reiter, Iris Scharnreitner, Horst Steiner, Johannes Zschocke, Mateja Pfeifer

**Affiliations:** Medical University of Graz Diagnostic and Research Institute of Human Genetics Neue Stiftingtalstr. 6 8010 Graz Austria; Graz Ragnitz Private Hospital Prenatal Center at Graz Berthold-Linder-Weg 15 8047 Graz Austria; Medical University of Graz D&R Institute of Human Genetics Neue Stiftingtalstr. 6 8010 Graz Austria; Paracelsus Medical University Department of Obstetrics and Gynecology Nonntaler Hauptstr. 55 5020 Salzburg Austria; Medical University of Graz Department of Obstetrics and Gynaecology, Research Unit for Foetal Medicine Auenbruggerplatz 14 8036 Graz Austria; Medical University Graz Department of Obstetrics and Gynaecology, Research Unit for Foetal Medicine Auenbruggerplatz 14 8036 Graz Austria; FetoMed-Fetal Medicine Clinic Heiligenstädter Str. 55–63 1190 Vienna Austria; Medical University of Graz D&R Institute of Human Genetics Neue Stiftingtalstr. 6 8010 Graz Austria; Medical University of Vienna Department of Obstetrics and Gynaecology Waehringer Str. 10 1090 Vienna Austria; Kepler University Hospital Medical Faculty, Institute of Medical Genetics Krankenhausstr. 9 4020 Linz Austria; Kepler University Hospital Medical Faculty, Institute of Medical Genetics Krankenhausstr. 9 4020 Linz Austria; praenamed GmbH Institute for Prenatal Diagnostics Nonntaler Hauptstr. 55 5020 Salzburg Austria; University of Innsbruck Division of Human Genetics Peter-Mayr-Str. 1 6020 Innsbruck Austria; Medical University of Vienna Institute of Medical Genetics Waehringer Str. 10 1090 Vienna Austria

**Keywords:** prenatal, diagnostics, genetic testing, guideline, recommendations

## Abstract

The increasing availability of chromosomal microarray (CMA), exome and genome analysis for prenatal diagnostic testing, together with concerns of potential legal consequences in cases of missed diagnoses, has contributed to substantial uncertainty in prenatal medicine. To support consistent and clinically meaningful use of genetic diagnostics in Austria, the working group for prenatal genetic diagnostics reviewed existing guidelines and recommendations and agreed on a consensus addressing eight key questions arising from clinical practice. Given the limited predictive value of genomic findings in structurally normal fetuses, the working group recommends a strictly phenotype-driven diagnostic approach with CMA, exome and genome analysis to be systematically offered in the presence of fetal pathologies.

## Introduction

Prenatal diagnostics were traditionally focused on the identification of chromosomal disorders and targeted single gene testing in the last decade. The widespread use of CMA and next-generation sequencing (NGS) analyses for the detection of rare genetic disorders has also found its way into prenatal medicine in recent years. However, consistently defined indications for the application of this powerful technique have not yet been fully established.

**Figure 1:**
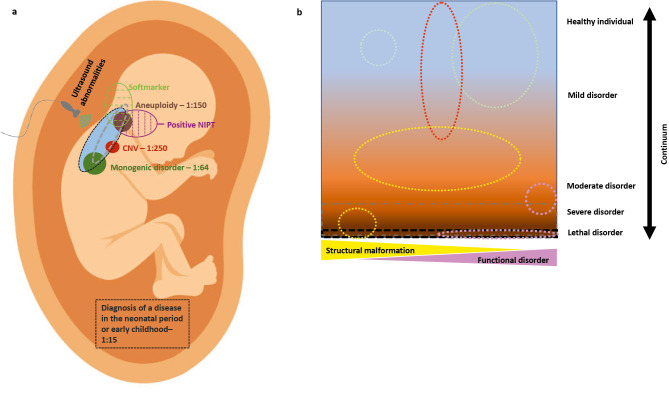
a) Prevalence of pathogenic genetic variants in the fetus (aneuploidy in brown, CNVs in red, monogenic disorders in green (prevalence 1:150, 1:250 and 1:64, respectively [5,6,7], estimation of actual illness perception and diagnosis in the neonatal period and childhood (prevalence of genetic and non-genetic disease 1:15, in light blue). Balancing the highest possible sensitivity for severe diseases, false positive results or true positive results which are not helpful in the prenatal setting. b) Variable expressivity and reduced penetrance of different genetic diseases (circles) pose a problem for predicting the possible course of the disease in an expected child.

Fetal abnormalities are detected with ultrasound examination in 3 % of pregnancies. A genetic cause is found in only a part of the affected fetuses, with the highest diagnostic yield of 53 % for skeletal dysplasia [1]. For many genetic disorders, there is still limited information on associated prenatal abnormalities, complicating prenatal diagnosis [2]. Genetic disorders are also found in about 1 % of fetuses without structural anomalies using chromosomal microarray analysis (CMA) and in about 2 % of fetuses using trio-whole exome sequencing (t-ES), with 0,9 % thereof leading to a severe disease [3]. A great part of genetic disorders listed in the OMIM (Online Mendelian Inheritance in Men) database are associated with incomplete penetrance and variable expressivity [4]. In these cases, it is not possible to predict whether the disease will actually manifest. This discrepancy must be considered when applying genetic diagnostics during pregnancy (Figure 1).

In cases of fetal abnormalities detected by ultrasound, pregnant women are offered different genetic testing strategies across Austrian clinics. This inconsistency not only leads to unequal management but also creates uncertainty among prenatal specialists and geneticists, who may fear legal consequences in cases of undiagnosed fetal conditions. Lack of a common strategy is also likely to lead to overdiagnosis in some children with subtle fetal findings such as isolated soft markers, which – especially when reporting low-penetrance variants – can also have negative consequences for the family. A structured, risk-adapted approach to invasive prenatal genetic testing is therefore essential. Indications must differentiate between fetuses with a high likelihood of a monogenic or chromosomal disorder, those with isolated findings of typically multifactorial origin, and constellations requiring targeted testing. Furthermore, the sequential and parallel use of rapid aneuploidy testing, chromosomal analysis, copy number variation (CNV) analysis, and exome-based approaches must be clearly defined to ensure rapid diagnosis.

This review of existing recommendations and literature served as the basis for an Austrian consensus conference of an interdisciplinary working group for prenatal genetic testing. The conclusions of the meeting on diagnostic prenatal testing including indication, test selection, tissue selection and analysis process with a main focus on CMA and NGS analysis are summarized.

## Material and Methods

### Review of relevant guidelines and recommendations

Guidelines on prenatal genetic testing were searched on homepages of scientific societies (ACMG, ASHG, ESHG, GfH, OEGH) and in PubMed using the terms “guideline”, “prenatal”, “exome” or “genome” and “genetic”. Results were restricted to those available in English or German and published between 2020 and 2025.

### Preparation of the consensus conference

The meeting of the Austrian working group for prenatal genetic testing was mainly prepared by participants and representatives of the executive board of the Austrian Society of Human Genetics and the Austrian Society for Ultrasound in Medicine. The following questions regarding genetic testing were distributed in advance and discussed by an interdisciplinary working group consisting of Austrian experts from the field of medical genetics and prenatal medicine:

1. Which genetic diagnostics should be offered in case of fetal ultrasound abnormalities?
2. Which genetic diagnostics should be offered in case of increased nuchal translucency?
3. Which genetic diagnostics should be offered in case of abnormal biochemistry results?
4. Which genetic diagnostics should be offered in the presence of “soft markers”?
5. Which genetic diagnostics should be offered when there is a familial risk for a genetic disorder in the fetus?
6. Should genetic diagnostics be offered in a pregnancy without detectable fetal anomalies?
7. Which tissues should be examined?
8. Should genetic analyses be performed sequentially or in parallel?

Established indications such as confirmatory genetic analyses after abnormal prenatal screening were not discussed.

A consensus was found for each of the questions, following a short presentation of scientific background and discussion of available international guidelines and recommendations in a meeting on 27^th^ of November 2025.

The conclusions were presented and discussed by a large auditorium at a conference for gynecological and prenatal ultrasound on 28^th^ November 2025 in Seggauberg (Styria, Austria).

## Results

### Risk assessment of genetic disorders in fetuses with and without detection of ultrasound anomalies

The rare disease database orphanet contains information on more than 6000 rare diseases. The cumulative point prevalence of rare diseases is 3.5–5.9 % [8]. Of all malformations detected in pregnancy, 27.6 % are detected in the 1^st^, 53.8 % in the 2^nd^ and 18.6 % in the 3^rd^ trimenon [9].

Chromosomal abnormalities are detected in about 0.7–2.5 % of newborns, depending on the maternal age [5]. Pathogenic CNVs are detected in about 0.4–1 % of normal fetuses [10, 11, 3], monogenic diseases are detected with a prevalence of 1.5–4 % [7, 12]. In a cohort of 316 fetuses with ultrasound anomalies, karyotype and CMA together had a diagnostic yield of 4.1 %. The additional diagnostic rate with t-ES was 15.8 % [13]. The additional diagnostic yield using genome analysis was about 1 % [14] (see Table 1 for prevalence of diseases). Diagnostic yields depend on the fetal phenotype [1], with skeletal phenotypes having the highest probability for receiving a diagnosis (53 %), followed by neuromuscular (37 %), multisystem (29 %), central nervous system (17 %), cardiac (11 %), craniofacial (9 %) and kidney/urinary tract anomalies (9 %) respectively. Some genetic disorders cannot be easily detected with these broad testing strategies, but need special genetic tests (Table 2).

**Table 1:** Prevalence of genetic disorders in newborns detected with chromosomal analysis, CMA, exome or genome analysis. 2^nd^ T=second trimester, ID=intellectual disability

Genetic disorder	(Average) Prevalence at birth	Citation
**Chromosomal abnormalities**	**1:150** 2^nd^ T 1:122→1:40, age 20→40y	[5]
Klinefelter syndrome (47,XXY)	**1:500**	[15]
Trisomy 21 (Down syndrome)	**1:700** 2^nd^ T 1:1.250→1:86, age 20→40y	[5, 16, 17]
Monosomy X (Turner syndrome)	**1:2.500**	[15, 17]
Trisomy 18 (Edwards syndrome)	**1:3.000** 2^nd^ T 1:5.000→1:333, age 20→40y	[16,17,18,19]
Trisomy 13 (Pätau syndrome)	**1:6.000** 2^nd^ T 1:10.000→1:714, age 20→40y	[15]
Pallister Killian syndrome, Isochromosome 12p mosaicism	**1:20.000**	[20]
**Copy number variants**	**1:250** 1:100 (in pregnancy)	[6, 10, 11]
**Monogenic diseases**	**1:27–1:64**	[7]
Newborn screening panel; disorders with interventions and neurodevelopmental delay incl. seizures Monogenic diseases in structurally normal fetuses (moderate/severe phenotype) Potentially significant variants	1:27 1:64 **(1:200 moderate/1:200 severe)** 1:37 **1:110 severe** (shortened life span, ID) 1:90 mild phenotype	[7] [12] [3]

**Table 2:** Examples of genetic disorders requiring special testing strategies. UPD: uniparental disomy.

Disease with special testing strategy	Prevalence	Possible findings on ultrasound examination	Citation
**Prader-Willi syndrome** (60–70 % deletion, 27–37 % UPD, 3 % epimutation)	**1:10.000–30.000**	Reduced fetal movements, polyhydramnios	[21, 22]
**Silver-Russell syndrome** (30–60 % epimutation, 5–10 % UPD)	**1:15.900**	Fetal growth retardation, macrocephaly, facial dysmorphism	[21, 23]
**Beckwith-Wiedemann syndrome** (55 % epimutation, 20 % UPD, 1 % chromosomal rearrangement, 20 % unknown)	**1:10.000–21.150**	Fetal overgrowth, omphalocele, placentomegaly, mesenchymal dysplasia	[21, 24]
**Angelman syndrome** (70 % deletion, 3–7 % UPD, 11 % *UBE3A* sequence variant)	**1:27.200**	-	[21]
**Congenital myotonic dystrophy 1** (DM1) Repeat expansion	**1:8.000** (including adult phenotype)	Polyhydramnios, reduced fetal movements	[25]
**Fragile X syndrome** Repeat expansion	**1:7.000–10.000**	-	[26]
**Spinal muscular atrophy, type** 1 (SMA type 1)	**1:6.000–8.000**	Reduced fetal movements	[27]

### Guidelines and recommendations for genetic testing methods

Ten relevant guidelines including prenatal genetic testing strategies were identified (see Table 3).

### 1 Which genetic diagnostics should be offered in case of fetal ultrasound abnormalities?

Most guidelines and statements recommend t-ES in case of ultrasound anomalies suggestive for a genetic disorder (SEGO, NHS, ACMG, SOGC, ISPD; see Table 1). The Spanish guidelines restrict the indication to a few defined fetal anomalies with an expected diagnostic yield of at least 20 % [31]. The German Society recommends CMA and/or gene panel or phenotype based virtual panel analysis using t-ES data, depending on the fetal phenotype and following the exclusion of aneuploidy. Phenotype-based virtual panel analysis is recommended to reduce incidental findings, while accepting a reduced sensitivity [29].

The ISPD recommends the use of t-ES for diagnostic sequencing. It is uncertain whether gene panel analysis with genes known to be associated with the phenotype, or a broader genome-wide analysis strategy is a better approach, as there is still limited knowledge about the correlation of genotype and the fetal phenotype[36].

The NHS recommends targeted analysis if sonographic findings indicate a specific monogenic disorder [33].

Prevalence estimations in large neurodevelopmental disease cohorts confirmed assumed heterogeneity of causes, with a prevalence of 0.3 % for disease-related *ARID1B* variants as the most common genetic cause. A gene panel of 100 genes with the highest prevalence is estimated to have a diagnostic yield of 8.8 %, while a panel with 500 genes yields <20 % compared to a diagnostic yield of 36 % for ES [37]. This shows that only a comprehensive testing strategy can accommodate this variability of genetic causes.

*Consensus: In the presence of fetal anomalies associated with a high likelihood of a genetic disorder, invasive testing including aneuploidy testing, chromosomal analysis, copy number analysis (CNV-analysis, e. g. CMA or next generation sequencing based CNV-analysis) and t-ES are indicated.*

*There is no indication for diagnostic invasive testing when an isolated condition with typically multifactorial aetiology is present (e. g., isolated neural tube defects/spina bifida, small muscular ventricular septal defect, gastroschisis, congenital pulmonal malformation/CPAM).*

*For highly specific suspected diagnoses, targeted genetic testing should be performed first. In cases of a specific suspicion of a group of disorders, exome-based virtual panel analysis may be sufficient (e. g., craniosynostosis).*

*Phenotype-based genetic analysis including copy number analysis may be performed via trio genome analysis (t-GS) instead of t-ES analysis.*

*We do not recommend using trio genome analysis as a routine analysis following normal exome results. However, we would like to point out, that differential diagnoses should be considered which cannot be detected by exome analysis (see Table 2).*

**Table 3:** Recommendations for genetic testing strategies in pregnancy

Recommendation	CMA, exome and genome analysis	First-tier genetic testing	other
**AWMF (2024)** [28]	No clear recommendations on the use of CMA, ES and GS. Molecular genetic testing (e. g. CMA or ES) should be offered if **NT ≥3.0 mm, but no later than >3.5 mm.**	Diagnostics for elevated NT: Cytogenetics (DP, PCR, FISH) → e. g. **CMA/t-ES**	
**GfH (2023)** [29]	CMA and/or phenotype-based t-ES for US anomalies using a virtual phenotype-generated gene panel to reduce incidential findings. ES without elevated risk for genetic disorders is not recommended.	After exclusion of aneuploidy (FISH/qPCR/QF-PCR) → **CMA and/or gene panel or ES**	t-ES analysis is recommended, diagnostics parallel or sequential, depending on week of gestation and available amount of DNA.
**FFGH** **(2024)** [30]	Prenatal ES should be prescribed by a doctor with expertise in the field of genetics and the field of fetal medicine. A multidisciplinary team discusses ES indication.	**CMA and ES should be performed** **in parallel** for faster diagnosis	If termination of pregnancy is decided due to fetal anomalies, postnatal ES/GS should be performed including fetal autopsy results.
**SEGO/AEDP (2024)** [31]	NGS diagnostic tests for single or multiple structural abnormalities with a suggestive pattern of monogenic origin (recommended for **expected diagnostic yield of 20 %**, considered for >10 %), not for other indications. Pregnancies with strong suspicion of recurrence risk for familial disorder (previous affected fetus/stillbirth/child – no sample for genetic testing of affected individuals available).	NGS mostly applied if no genetic alterations have been identified with QF-PCR and CMA. **NGS may also be applied as first-tier genetic test to assess fetal phenotypes highly suggestive of other monogenic anomalies.**	CNV analysis in panel, exome and genome analysis is considered as screening test, should be confirmed with additional technique (karyotyping, FISH, CMA, MLPA, digital PCR). Trio-analysis is preferred to singleton analysis.
**SIGO (2024)** [32]	CMA is recommended due to increased risk for fetal aneuploidies. ES or other NGS methods are not mentioned.	Diagnostics due to increased aneuploidy risk → rapid testing /QF-PCR, FISH) or CMA + standard karyotype	
**NHS** **(2025)** [33]	CMA and t-ES for panel analysis is indicated in a fetus with major structural abnormalities in an ongoing pregnancy. Trio-GS is indicated, if the pregnancy is not ongoing.	Rapid aneuploidy testing→ CMA** and ES in parallel**	Targeted testing if familial variants are known or if sonographic findings indicate a specific monogenic disorder.
**ACMG (2020)** [34]	ES may be considered for a fetus with one or more significant US anomalies. No data support the use of ES for other reproductive indications such as markers for aneuploidy or recurrent pregnancy loss.	CMA and karyotype. Targeted genetic analysis is recommended, if a specific diagnosis is suspected.	Trio analysis is preferred over singleton analysis.
**ACOG (2020)** [17]	Patients who prefer comprehensive prenatal testing, should be offered CMA and “diagnostic testing”. **Diagnostic testing (not specified)** should be offered for **NT ≥ 3mm** or > 99p, cystic hygroma in FTS, mild to moderate ventriculomegaly in second trimester.	Screening and diagnostic testing for chromosomal anomalies should be offered to all patients early in pregnancy regardless of maternal age or baseline risk.	Pretest counselling: family history including birth defects, intellectual disability, genetic diagnoses and multiple miscarriages may influence testing decisions.
**SOGC (2024)** [35]	If a fetal structural abnormality is identified in the first or second trimester US, CMA or ES/GS is recommended.	Rapid aneuploidy detection → **CMA or ES/GS analysis**	
**ISPD (2022)** [36]	ES/GS is only recommended in fetuses with **major single anomaly OR multiple organ system anomalies AND if the phenotype is suggestive of a genetic etiology,** no other indication. Exception: recurrent childhood-onset severe genetic condition without prenatal phenotype in previous children for whom no genetic testing was done and possible.	CMA. **If a multiple anomaly pattern strongly suggests a monogenic disorder, ES can be the first line diagnostic method.**	As prenatal ES is not currently validated to detect CNVs, CMA should be run before or in parallel. Trio analysis is preferred over singleton analysis.

ACMG – American College f Medical Genetics and Genomics ACOG – American College of Obstetricians and Gynecologists AEDP – Asociación Española de Diagnóstico Prenatal (Spanish Association of Prenatal Diagnosis) AWMF – Arbeitsgemeinschaft der Wissenschaftlichen Medizinischen Fachgesellschaften FFGH – French Federation of Human Genetics Working Group GfH – German Society of Human Genetics ISPD – International Society for Prenatal Diagnosis NHS – National Health Service England – (National Genomic Test Directory) SEGO – Spanish Society of Gynecology and Obstetrics SIGO – Italian Society for Obstetrics and Gynecology SOGC – Society of Obstetricians and Gynaecologists of Canada CMA = chromosomal microarray analysis, CNV = copy-number variation, (t-)ES = (trio-) exome sequencing, DP=digital PCR, QF-PCR=quantitative fluorescent PCR, FTS = first trimester screening, GS = genome sequencing, NGS = next-generation sequencing, NT = nuchal translucency, p = percentile, UPD = uniparental disomy, US = ultrasound.

### 2 Which genetic diagnostics should be offered in case of increased nuchal translucency?

Ultrasound measurement of nuchal translucency (NT) thickness is part of the combined first-trimester screening. Combined first-trimester screening is used to calculate risks for the common aneuploidies, i. e., trisomies 21, 18 and 13.

An increased nuchal translucency is not considered a specific marker for the frequent trisomies alone. It also represents a risk factor for other chromosomal abnormalities, genetic syndromes (e. g. Rasopathies) and malformations. Consequently, further molecular genetic testing is recommended when cytogenetic analysis yields normal results.

There is ongoing debate regarding the threshold at which trisomy 21 risk calculation should be omitted and direct invasive testing pursued. Proposed cut-offs include the 95^th^ and the 99^th^ percentile, as well as 3.0 mm and 3.5 mm. However, it is a general agreement that, in the presence of structural anomalies or an NT measurement exceeding 3.5 mm, diagnostic invasive testing should be offered. If the cytogenetic analysis is unsuspicious, molecular genetic testing (e. g. CMA, t-ES) should be offered.

Several studies have investigated the diagnostic yield of genetic tests using different NT cut-off values. However, studies focusing on fetuses with isolated increased NT are limited by small sample sizes, while larger cohorts often fail to differentiate between isolated increased NT and those accompanied by additional structural anomalies, limiting the interpretation and generalizability of their findings.

Maya et al. recommended performing CMA analysis for NT measurements ≥3.0 mm in the absence of additional structural anomalies. They compared the proportion of pathogenic variants across three groups: <3.0 mm (n=462), 3.0–3.4 mm (n=170), and >3.4 mm (n=138). The rate of chromosomal abnormalities increased from 1.7 % to 6.5 % and 13.8 %, respectively. Furthermore, pathogenic variants detectable only by CMA, but not by conventional cytogenetics or cell-free DNA (cfDNA) testing, were found in 0.9 %, 1.8 %, and 2.2 % of cases, respectively [38].

In a systematic review by Giralomo et al., fetuses with isolated increased NT ≥3.0 mm but normal standard karyotype and CMA analysis were found to have pathogenic or likely pathogenic variants in 8 % of cases detected exclusively by ES. When considering fetuses with isolated NT and normal fetal anatomy at the anomaly scan, the rate of pathogenic or likely pathogenic variants detected by ES was 3,87 % [39].

So far, there are no studies investigating the frequency of pathogenic or likely pathogenic variants detected by ES in a subgroup of fetuses with isolated nuchal translucency of 3.0–3.5 mm.

*Consensus: For a NT measurement of ≥3.0 mm a rapid aneuploidy test/chromosomal analysis and CNV-analysis are indicated. If structural anomalies are suspected during first-trimester screening and/or NT is >3.5 mm, t-ES should additionally be offered. An early anatomical scan may be helpful for further assessment of diagnostic indications.*

### 3 Which genetic diagnostics should be offered in cases of abnormal biochemistry?

The Austrian, German and Swiss Societies (AWMF Guideline) recommend offering a molecular genetic analysis for abnormally low levels of PAPP-A or ß-HCG or high ß-HCG values in a maternal blood sample [28]. The working group endorses this recommendation and further specifies that CNV-analysis should be offered, but not t-ES, as there is currently no scientific evidence to support it.

*Consensus: If PAPP-A and/or free β-hCG are reduced (<0.2 MoM), or if free β-hCG is elevated (>5.0 MoM), rapid aneuploidy test/chromosomal analysis and CNV-analysis are indicated.*

### 4 Which genetic diagnostics should be offered in the presence of “soft markers”?

Soft markers are common ultrasonographic findings observed during routine midtrimester ultrasound examination. They are nonspecific and often represent minor transient anatomic variations (e. g. nuchal translucency, cardiac echogenic focus, hypoplastic nasal bone, short femur/humerus, mild hydronephrosis (4–7mm) in the second trimester, hyperechogenic bowels, aberrant right subclavian artery (ARSA)). Historically, the presence or absence of specific soft markers was used to assess the probability of trisomy 21 for high-risk pregnancies. However, this approach has largely been replaced by more accurate prenatal screening methods. cfDNA is the single best screening test for the common trisomies (trisomies 21, 18, and 13). Consequently, ultrasound screening for trisomy 21 based solely on soft markers is no longer the method of choice. Therefore, when an isolated soft marker is present, a cfDNA test may be recommended.

Beyond aneuploidy, soft markers have also been associated with pathogenic CNVs. In a study by Pan et al., 6.43 % of fetuses with soft markers in the second trimester were found to have genetic alterations (3.04 % aneuploidy, 3.4 % CNVs). The likelihood of detecting a genetic abnormality increased with the number of soft markers. Among isolated soft markers, an absent or a hypoplastic nasal bone in the second trimester showed the highest diagnostic yield for aneuploidy of 5.22 %, with an additional 2.95 % rate of pathogenic or likely pathogenic CNVs [40].

Certain findings traditionally categorized as soft markers may represent structural anomalies with a higher likelihood of an underlying genetic disorder. These include the absence of the nasal bone, ventriculomegaly and a shortening of long bones below the 3^rd^ percentile. This distinction is clinically relevant, as structural abnormalities justify comprehensive genetic testing strategies, whereas isolated soft markers without additional findings generally do not.

*Consensus: Screening for trisomy 21 based solely on soft markers is no longer the method of choice (NIPT provides superior sensitivity). The presence of an isolated soft marker is* ***not**** an indication for CNV-analysis and/or ES.*

*When* ***≥2 soft markers**** are present, rapid aneuploidy test/chromosomal analysis and CNV-analysis are indicated.*

***Exceptions are markers considered as structural anomalies:***

*In cases of ventriculomegaly or a short femur (<3^rd^ percentile), together with shortening of the long bones detected in the second or third trimester, CNV-analysis and t-ES are indicated in addition to rapid aneuploidy test and chromosomal analysis.*

*For an isolated absent nasal bone, chromosomal analysis and copy number analysis/CMA are indicated. If the fetal profile is abnormal with suspicion of a genetic syndrome or if additional malformations are present, CMA/copy number analysis and t-ES are indicated.*

### 5 Which genetic diagnostics should be offered when genetic variants are known in the family?

The NHS and ACMG recommend targeted testing to exclude known familial genetic variants [33, 34].

*Consensus: In cases of an increased familial risk for a severe fetal disorder or a childhood-onset disease due to one or more known familial disease-causing variant(s) (carrier status of parents or affected siblings), targeted testing is indicated. In addition, chromosomal analysis should be offered.*

### 6 Should genetic diagnostics be offered without a fetal indication?

The ACMG states that prenatal testing strategies should be individualized and guided by prenatal imaging findings and family history [34]. The ACOG recommends that chromosomal analysis should be offered to all pregnant women, regardless of maternal age or baseline risk [17]. This offer cannot however be seen as diagnostic, but rather as a screening option, if the pregnancy and the fetus are inconspicuous. The German Society of Human Genetics and the International Society for Prenatal Diagnosis recommend against performing t-ES in the absence of a clear fetal risk constellation [29, 36].

*Consensus: There is no indication for CNV-analysis, t-ES, or GS in the absence of a high risk for a genetic disorder, even at the parents’ request.*

*If a sample is already available due to other investigations (e. g., infection screening, testing for known familial variants), sending it to a genetics laboratory for DNA storage should be offered.*

*A chromosomal analysis should be additionally offered (maintenance of established practice).*

### 7 Which tissue should be examined?

Chorionic villus sampling (CVS) is possible from week 11+0, providing cytotrophoblast and extraembryonic mesodermal cells from the fetal part of the placenta. Placental mosaicism can lead to a false positive or false negative result and is estimated to occur in 1–2 % of CVS samples [41]. 13 % of mosaics are true fetal (chromosomes 13, 18, 21, 9, 15, 16, 22 have an increased rate of meiotic origin) [42].

Cytotrophoblast cells are analysed preferentially in direct preparation of the CVS sample. Mesenchymal cells may predominate in a cultured sample. While both cultures can display false positive results, false negative results are rarely seen. Trisomy or monosomy not seen in the fetus but in the CVS sample can be a result of fetal trisomy or monosomy rescue, possibly leading to uniparental disomy and a fetal imprinting disorder [41].

Amniocentesis (AC) can be performed from week 15+0 and provides fetal cells shed into amniotic fluid [41]. Therefore, suspected fetal mosaicism (e. g. Pallister-Killian syndrome) can be more reliably detected with AC. Exclusion of suspected chromosomal aberration due to cfDNA test in a pregnancy with a normal fetus is best performed with AC [43], but suspected mosaicism can never be completely ruled out. True mosaicism occurs in 0.2 % of AC samples (generally involving sex chromosomes, or chromosomes 21, 18, 13). 0.76 % reveal multiple cell pseudo mosaicism and 3.73 % single cell pseudo mosaicism[42].

*Consensus: The goal of prenatal genetic diagnostics is to achieve the most rapid diagnosis possible. The choice between CVS and AC should be guided by the time at which ultrasound abnormalities are detected.*

*Exceptions:*

*Targeted molecular genetic testing for familial genetic diseases should generally be performed as early as possible using CVS.*
*Confirmation of an abnormal NIPT result should, in cases where a false-positive result is suspected (e. g., normal fetal anatomy despite suspected trisomy 13 or 18), be performed by AC (fetal cells; note the risk of placental mosaicism).*
*If an imprinting disorder is suspected, AC is preferred (due to the development of methylation patterns during pregnancy).*
*In cases of intrauterine fetal demise or planned termination of pregnancy, DNA should be obtained via AC/CVS whenever possible, or through rapid post-delivery tissue sampling from the fetus or placenta, in order to ensure the highest possible DNA quality.*

### 8 Should genetic analyses be performed sequentially or in parallel?

The French Guidelines [30] and the NHS [33] concluded that a sequential testing strategy delays the final genetic result, increasing the stress of the parents in pregnancy. Considering the highest interest of the pregnant woman and couples as well as economical and organizational aspects, it was decided to perform genetic analyses in parallel. In line with these guidelines, the working group emphasized the need for a genetic diagnosis in the prenatal setting.

*Consensus: Exclusion of maternal cell contamination, rapid aneuploidy test, or alternatively chromosomal analysis from the short-term culture of CVS, should be available before proceeding with further genetic analyses. If aneuploidy testing is normal, it is not necessary to wait for the chromosomal analysis result before initiating additional testing. If targeted diagnostics are indicated, the results of the specific tests should be obtained before initiating comprehensive analyses (CNV-analysis and t-ES).*

*Sequential processing of the required examinations should not cause any delay in diagnostics during an ongoing pregnancy (parallel evaluation of CNV-analysis and t-ES).*

*In cases of planned pregnancy termination due to ultrasound abnormalities or intrauterine fetal demise (IUFD), the fetal pathology results should be awaited and considered in the diagnostic process.*

***Additional questions from the presentation of the consensus at the conference for gynecological and prenatal ultrasound on 28th of November 2025 in Seggauberg (Styria, Austria)***

**Why can’t we offer t-ES to all pregnant women, regarding the diagnostic rate in structurally normal foetuses?**

This decision was justified by the fact that the aim of the working group was to develop guidelines for prenatal genetic diagnostics, not for prenatal genetic screening. If t-ES would be offered in every pregnancy with the current evaluation strategy, we would detect many variants in presumably healthy fetuses whose clinical significance could not be predicted. For genetic screening using t-ES, a meaningful evaluation strategy would first have to be developed.

## Discussion

Genetic disorders can be detected in a relatively high percentage of structurally normal fetuses when broad diagnostic methods are applied. The difficulty in clinical management in these cases arises from the fact that, for many conditions, high variability and incomplete penetrance have been described. Whether the fetus will develop the disease later in life, and what the course of the disease will be, might not be predicted for the individual fetus. The question remains what consequences such genetic findings have during pregnancy. We are very aware of the limitations of knowledge regarding prenatal phenotypes and rare diseases, and we therefore accept that the interpretation of genetic results is challenging. On the other hand, the goal of prenatal medicine is to achieve the highest possible diagnostic rate with regard to severe fetal and childhood diseases. Expectant parents should get the opportunity for early diagnosis of serious fetal conditions to have the option to adjust clinical care and prepare for the condition or to terminate the pregnancy. The challenges here lie in offering the most comprehensive diagnostics possible in order to also detect rare diseases, complex genetic variants (Table 3), or mosaics while minimizing uncertainty caused by findings that are difficult to interpret. A clear distinction between mild, moderate, and severe disease courses in relation to genetic variants is not fully possible.

A very important part of pre-test counselling is also the family history including history of birth defects, intellectual disability, genetic diagnoses and multiple miscarriages in family members [17]. This information can help to guide the diagnostic prenatal steps. In cases of a known disease-causing familial variant or a high suspicion of a specific genetic disorder, a targeted analysis should be offered.

## Conclusion

The review of international guidelines and recommendations revealed inconsistent practices in the field of prenatal diagnostics. To harmonize the genetic testing strategies in Austria, an eight-point statement on prenatal genetic testing has been elaborated by the Austrian working group for prenatal genetic testing.

## References

[1] Mellis R, Oprych K, Scotchman E, Hill M, Chitty LS (2022). Diagnostic yield of exome sequencing for prenatal diagnosis of fetal structural anomalies: A systematic review and meta-analysis. Prenat Diagn 42(6).

[2] Petrovski S, Aggarwal V, Giordano JL, Stosic M, Wou K, Bier L, Spiegel E, Brennan K, Stong N, Jobanputra V, Ren Z, Zhu X, Mebane C, Nahum O, Wang Q, Kamalakaran S, Malone C, Anyane-Yeboa K, Miller R, Levy B, Goldstein DB, Wapner RJ (2019). Whole-exome sequencing in the evaluation of fetal structural anomalies: a prospective cohort study. Lancet 393(10173).

[3] Levy M, Lifshitz S, Goldenberg-Fumanov M, Bazak L, Goldstein RJ, Hamiel U, Berger R, Lipitz S, Maya I, Shohat M (2025). Exome sequencing in every pregnancy? Results of trio exome sequencing in structurally normal fetuses. Prenat Diagn 45(3).

[4] (12/01/2025). Online Mendelian Inheritance in Man, OMIM®. Online Mendelian Inheritance in Man, OMIM®.

[5] Nussbaum RL, McInnes RR, Willard HF (2016). Principles of clinical cytogenetics and genome analysis. Thompson & Thompson genetics in medicine. 8th ed.

[6] Srebniak MI, Joosten M, Knapen MFCM, Arends LR, Polak M, van Veen S, Go ATJI, Van Opstal D (2018). Frequency of submicroscopic chromosomal aberrations in pregnancies without increased risk for structural chromosomal aberrations: systematic review and meta-analysis. Ultrasound Obstet Gynecol 51(4).

[7] Ziegler A, Koval-Burt C, Kay DM, Suchy SF, Begtrup A, Langley KG, Hernan R, Amendola LM, Boyd BM, Bradley J, Brandt T, Cohen LL, Coffey AJ, Devaney JM, Dygulska B, Friedman B, Fuleihan RL, Gyimah A, Hahn S, Hofherr S, Hruska KS, Hu Z, Jeanne M, Jin G, Johnson DA, Kavus H, Leibel RL, Lobritto SJ, McGee S, Milner JD, McWalter K, Monaghan KG, Orange JS, Pimentel Soler N, Quevedo Y, Ratner S, Retterer K, Shah A, Shapiro N, Sicko RJ, Silver ES, Strom S, Torene RI, Williams O, Ustach VD, Wynn J, Taft RJ, Kruszka P, Caggana M, Chung WK (2025). Expanded Newborn Screening Using Genome Sequencing for Early Actionable Conditions. JAMA 333(3).

[8] Nguengang Wakap S, Lambert DM, Olry A, Rodwell C, Gueydan C, Lanneau V, Murphy D, Le Cam Y, Rath A (2019). Estimating cumulative point prevalence of rare diseases: analysis of the Orphanet database. Eur J Hum Genet28(2).

[9] Syngelaki A, Hammami A, Bower S, Zidere V, Akolekar R, Nicolaides KH (2019). Diagnosis of fetal non-chromosomal abnormalities on routine ultrasound examination at 11–13 weeks’ gestation. Ultrasound Obstet Gynecol 54(4).

[10] Stern S, Hacohen N, Meiner V, Yagel S, Zenvirt S, Shkedi-Rafid S, Macarov M, Valsky DV, Porat S, Yanai N, Frumkin A, Daum H (2021). Universal chromosomal microarray analysis reveals high proportion of copy-number variants in low-risk pregnancies. Ultrasound Obstet Gynecol 57(5).

[11] Levy B, Wapner R (2018). Prenatal diagnosis by chromosomal microarray analysis. Fertil Steril 109(2).

[12] Sotiriadis A, Demertzidou E, Ververi A, Tsakmaki E, Chatzakis C, Mone F (2025). Incremental yield of exome sequencing over standard prenatal testing in structurally normal fetuses: systematic review and meta-analysis. Ultrasound Obstet Gynecol 65(5).

[13] Zeng Z, Zhang L, Zhou Y, Zhang X, Yi H, Li H, Liu Y, Li J, Chen Q, Chen Y, Yu G, Yi J, Zhang Y, Zhang H, Dong Y (2025). Clinical utility of trio whole exome sequencing in fetuses with ultrasound anomalies. Hum Genomics 19(1).

[14] Shreeve N, Sproule C, Choy KW, Dong Z, Gajewska-Knapik K, Kilby MD, Mone F (2024). Incremental yield of whole-genome sequencing over chromosomal microarray analysis and exome sequencing for congenital anomalies in prenatal period and infancy: systematic review and meta-analysis. Ultrasound Obstet Gynecol 63(1).

[15] Hook EB, Warburton D (1983). The distribution of chromosomal genotypes associated with Turner’s syndrome: livebirth prevalence rates and evidence for diminished fetal mortality and severity in genotypes associated with structural X abnormalities or mosaicism. Hum Genet 64(1).

[16] Mai CT, Isenburg JL, Canfield MA, Meyer RE, Correa A, Alverson CJ, Lupo PJ, Riehle-Colarusso T, Cho SJ, Aggarwal D, Kirby RS (2019). National Birth Defects Prevention Network. National population-based estimates for major birth defects, 2010–2014. Birth Defects Res. 111(18).

[17] (2020). Screening for Fetal Chromosomal Abnormalities: ACOG Practice Bulletin, Number 226. Obstet Gynecol 136(4).

[18] Savva GM, Walker K, Morris JK (2010). The maternal age-specific live birth prevalence of trisomies 13 and 18 compared to trisomy 21 (Down syndrome). Prenat Diagn 30(1).

[19] Springett A, Wellesley D, Greenlees R, Loane M, Addor MC, Arriola L, Bergman J, Cavero-Carbonell C, Csaky-Szunyogh M, Draper ES, Garne E, Gatt M, Haeusler M, Khoshnood B, Klungsoyr K, Lynch C, Dias CM, McDonnell R, Nelen V, O’Mahony M, Pierini A, Queisser-Luft A, Rankin J, Rissmann A, Rounding C, Stoianova S, Tuckerz D, Zymak-Zakutnia N, Morris JK (2015). Congenital anomalies associated with trisomy 18 or trisomy 13: A registry-based study in 16 European countries, 2000–2011. Am J Med Genet A 167A(12).

[20] (05/24/2021). Online Mendelian Inheritance in Man, OMIM®. Online Mendelian Inheritance in Man, OMIM®.

[21] Yakoreva M, Kahre T, Žordania R, Reinson K, Teek R, Tillmann V, Peet A, Õiglane-Shlik E, Pajusalu S, Murumets Ü, Vals MA, Mee P, Wojcik MH, Õunap K (2019). A retrospective analysis of the prevalence of imprinting disorders in Estonia from 1998 to 2016. Eur J Hum Genet 27(11).

[22] Driscoll DJ, Miller JL, Cassidy SB (1998 Oct 6). Prader-Willi Syndrome. GeneReviews®.

[23] Gambadauro A, Chirico V, Galletta F, Gulino F, Chimenz R, Serraino G, Rulli I, Manganaro A, Gitto E, Marseglia L Imprinting Disorders and Epigenetic Alterations in Children Conceived by Assisted Reproductive Technologies: Mechanisms, Clinical Outcomes, and Prenatal Diagnosis (2025). Genes (Basel) 16(10).

[24] Shuman C, Kalish JM, Weksberg R (2000 Mar 3). Beckwith-Wiedemann Syndrome. GeneReviews®.

[25] (07/17/2025). Online Mendelian Inheritance in Man, OMIM®. Online Mendelian Inheritance in Man, OMIM®.

[26] Hunter J, Rivero-Arias O, Angelov A, Kim E, Fotheringham I, Leal J (2014). Epidemiology of fragile X syndrome: a systematic review and meta-analysis. Am J Med Genet A 164A(7).

[27] (06/17/2025). Online Mendelian Inheritance in Man, OMIM®. Online Mendelian Inheritance in Man, OMIM®.

[28] von Kaisenberg C, Kozlowski P, Kagan KO, Hoopmann M, Heling KS, Chaoui R, Klaritsch P, Pertl B, Burkhardt T, Tercanli S, Frenzel J, Mundlos C (2024). Firsttrimester Diagnosis and Therapy @ 11-13 +6 Weeks of Gestation – Part 1: Guideline of the DEGUM, ÖGUM, SGUMGG, DGGG, ÖGG, Gynecologie Suisse, DGPM, DGPGM, BVF, ACHSE (AWMF S2e LL 085–002 1.1.2024). Geburtshilfe Frauenheilkd 84(10).

[29] (23.10.2023). Stellungnahme der Deutschen Gesellschaft für Humangenetik zum Umfang der genetischen Pränataldiagnostik bei auffälligen Ultraschallbefunden. Stellungnahme der Deutschen Gesellschaft für Humangenetik zum Umfang der genetischen Pränataldiagnostik bei auffälligen Ultraschallbefunden.

[30] Cogan G, Troadec MB, Devillard F, Saint-Frison MH, Geneviève D, Vialard F, Rial-Sebbag E, Héron D, Attie-Bitach T, Benachi A, Saugier-Veber P (2025). Use of Prenatal Exome Sequencing: Opinion Statement of the French Federation of Human Genetics Working Group. Prenat Diagn 45(3).

[31] Abulí A, Antolín E, Borrell A, Garcia-Hoyos M, García Santiago F, Gómez Manjón I, Maíz N, González González C, Rodríguez-Revenga L, Valenzuena Palafoll I, Suela J (2024). Guidelines for NGS procedures applied to prenatal diagnosis by the Spanish Society of Gynecology and Obstetrics and the Spanish Association of Prenatal Diagnosis. J Med Genet 61(8).

[32] Stampalija T, Ghi T, Barbieri M, Morlando M, Di Pasquo E, Formigoni C, Ferrazzi E (2024). The Italian guidelines on non-invasive and invasive prenatal diagnosis: Executive summary of recommendations for practice the Italian Society for Obstetrics and Gynecology (SIGO). Eur J Obstet Gynecol Reprod Biol 300.

[33] (July 2025). Testing Criteria for Rare and Inherited Disease. Testing Criteria for Rare and Inherited Disease.

[34] Monaghan KG, Leach NT, Pekarek D, Prasad P, Rose NC (2020). The use of fetal exome sequencing in prenatal diagnosis: a points to consider document of the American College of Medical Genetics and Genomics (ACMG). Genet Med 22(4).

[35] Audibert F, Wou K, Okun N, De Bie I, Wilson RD (2024). Guideline No. 456: Prenatal Screening for Fetal Chromosomal Anomalies. J Obstet Gynaecol Can 46(11).

[36] Van den Veyver IB, Chandler N, Wilkins-Haug LE, Wapner RJ, Chitty LS (2022). International Society for Prenatal Diagnosis Updated Position Statement on the use of genomewide sequencing for prenatal diagnosis. Prenat Diagn 42(6).

[37] Gillentine MA, Wang T, Eichler EE (2022). Estimating the Prevalence of De Novo Monogenic Neurodevelopmental Disorders from Large Cohort Studies. Biomedicines 10(11).

[38] Maya I, Yacobson S, Kahana S, Yeshaya J, Tenne T, Agmon-Fishman I, Cohen-Vig L, Shohat M, Basel-Vanagaite L, Sharony R (2017). Cut-off value of nuchal translucency as indication for chromosomal microarray analysis. Ultrasound Obstet Gynecol 50.

[39] Girolamo RD, Rizzo G, Khalil A, Alameddine S, Lisi G, Liberati M, Novelli A, D’Antonio F (2023). Whole exome sequencing in fetuses with isolated increased nuchal translucency: a systematic review and meta-analysis. The Journal of Maternal-Fetal and Neonatal Medicine 36:1.

[40] Pan L, Wu J, Liang D, Yuan J, Wang J (2023). Association analysis between chromosomal abnormalities and fetal ultrasonographic soft markers based on 15,263 fetuses. Am J Obstet Gynecol MFM 5.

[41] Bianchi DW, Wilkins-Haug LE, Enders AC, Hay ED (1993). Origin of extraembryonic mesoderm in experimental animals: relevance to chorionic mosaicism in humans. Am J Med Genet 46(5).

[42] Levy B, Hoffmann ER, McCoy RC, Grati FR (2021). Chromosomal mosaicism: Origins and clinical implications in preimplantation and prenatal diagnosis. Prenat Diagn 41(5).

[43] Wojcik MH, Reimers R, Poorvu T, Agrawal PB (2020). Genetic diagnosis in the fetus. J Perinatol 40(7).

